# Two new species of Polleniidae (Diptera) from China

**DOI:** 10.3897/zookeys.1269.172931

**Published:** 2026-02-13

**Authors:** Tong Fu, Yawen Xue, Jianxin Cui

**Affiliations:** 1 Breeding Research Center of Insect Pest’s Natural Enemies, Henan Institute of Science and Technology, Xinxiang, Henan 45003, China Breeding Research Center of Insect Pest’s Natural Enemies, Henan Institute of Science and Technology Xinxiang China; 2 Henan International Joint Laboratory of Taxonomy and Systematic Evolution of Insecta, Henan Institute of Science and Technology, Xinxiang, Henan 45003, China Henan International Joint Laboratory of Taxonomy and Systematic Evolution of Insecta, Henan Institute of Science and Technology Xinxiang China

**Keywords:** Cluster flies, digital illustration, identification key, *
Morinia
*, *
Pollenia
*, species key, taxonomy

## Abstract

*Morinia zhenhang* Fu, Xue & Cui, **sp. nov**. (振航墨粉蝇) collected from Beijing, China, and *Pollenia yuensis* Fu, Xue & Cui, **sp. nov**. (豫粉蝇) collected from Henan Province and Beijing, China, are described and illustrated. Keys to the Chinese males of the genus *Morinia* and to the Chinese species of the genus *Pollenia* are provided. Digital illustration techniques were used to depict the habitus, frontal head views, and terminalia of the two species. The type specimens are preserved in the Insect Museum of Henan Institute of Science and Technology and the Shanghai Entomological Museum, Chinese Academy of Sciences.

## Introduction

The family Polleniidae, formerly treated as a subfamily within Calliphoridae, has recently been elevated to family rank (Cerretti et al. 2019), with a generic key and a world checklist provided by Gisondi et al. (2020). Gisondi et al. (2023) proposed dividing Polleniidae into two subfamilies: Polleniinae and Moriniinae. To date, the family comprises 151 species in six genera worldwide (Xue et al. 2020; Schluesslmayr and Sivell 2021; Xue and Du 2022; Liu et al. 2025).

Polleniinae includes three genera. *Pollenia* is by far the most species-rich and widespread genus of the family, with 97 species mainly distributed in the Palaearctic, Oriental, and Australasian regions, and a few species introduced into the Afrotropical and Nearctic regions; nine species are currently recorded from China (Gisondi et al. 2020; Xue et al. 2020; Schluesslmayr and Sivell 2021). In addition to its remarkable diversity, *Pollenia* is the type genus of the family and the only genus for which substantial biological information is available, as several species are parasitoids of earthworms. *Dexopollenia* comprises 23 species distributed in the southeastern Palaearctic and Oriental regions, with 12 species recorded from China, while *Xanthotryxus* is restricted to China, with eight described species (Gisondi et al. 2020; Liu et al. 2025).

Moriniinae includes three genera. *Morinia* comprises 14 species distributed in the Afrotropical, Oriental, and Palaearctic regions, with five species recorded from China (Gisondi et al. 2020; Xue et al. 2020; Xue and Du 2022); *Melanodexia* includes eight species restricted to the USA; and *Alvamaja* is a monotypic genus from Romania and Serbia (Gisondi et al. 2020).

In this study, we describe two new species, *M. zhenhang* sp. nov. and *P. yuensis* sp. nov. and provide a key to Chinese males of *Morinia* and a key to the species of *Pollenia* known from China.

## Material and methods

Specimens were examined under a Motic K-400 stereomicroscope. High-resolution photographs were taken using a Leica M205FCA stereomicroscope fitted with an Imaging Source CCD camera. For examination of terminalia, the abdomens were detached and treated in warm 10% sodium hydroxide (NaOH) solution to macerate soft tissues. The material was then neutralized with acetic acid, after which the male and female terminalia, as well as male sternite 5, were dissected with fine forceps and stored in absolute ethanol. These structures were subsequently photographed using the same imaging setup. Final plates were prepared from the photographs using graphics software (Easy Paint Tool SAI, SYSTEMAX Inc., Tokyo, Japan; Adobe Photoshop 2019, Adobe Inc., California, USA; and a GAOMON 1060PRO graphics tablet, GAOMON Technology Co., Guangzhou, China). Color illustrations were produced by Tong Fu. Morphological terminology follows Xue and Du (2022), Cumming and Wood (2017), Fan (1997), and McAlpine (1981).

Abbreviations used are as follows: **a s**–anterior setae; **ac s**–acrostichal setae; **ad s**–anterodorsal setae; **av s**–anteroventral setae; **dc s**–dorsocentral setae; **fr s**–frontal setae; **ia s**–intra-alar setae; **iv s**–inner vertical setae; **kept s**–kepisternal setae; **npl s**–notopleural setae; **p s**–posterior setae; **pal s**–postalar setae; **pavt s**–paravertical setae; **pc orb s**–proclinate orbital setae; **pd s**–posterodorsal setae; **ph s**–posthumeral setae; **poc s**–postocellar setae; **povt s**–postvertical setae; **pprn s**–postprontal setae; **pr s**–presutural setae; **pra s**–prealar setae; **prepm s**–proepimeral setae; **prepst s**–proepisternal setae; **pv s**–posteroventral setae; **sa s**–supra-alar setae; **sc s**–scutellar setae; **v**–ventral setae; **ST**–abdominal sternites; **T**–abdominal tergites.

All material is deposited in both the Insect Museum of Henan Institute of Science and Technology (**HIST**) and the Shanghai Entomological Museum, Chinese Academy of Sciences (**SEMCAS**).

Type specimens were examined for comparison at SEMCAS and the Insect Collection of Shenyang Normal University (**SYNU**).

## Results

### Taxonomy

#### Genus Morinia

Taxon classificationAnimaliaDipteraPolleniidae

Robineau-Desvoidy, 1830

5C1E957D-1B9A-5CB9-8B24-0E6C2B88F4E6

Morinia Robineau-Desvoidy, 1830: 264. Type species: Morinia velox Robineau-Desvoidy, 1830 [= Musca doronici Scopoli, 1763], by subsequent designation (Rondani 1862: 159).

##### Diagnosis.

Small to medium-sized, slender flies, black in ground colour, rarely showing metallic bronze sheen or uniformly covered with grey pruinosity; outer ph s and presutural ia s absent; posterior thoracic spiracle small, circular, with anterior and posterior lappets subequal in size (posterior slightly larger) and directed outward; lower calypter narrow, diverging from scutellum; node at base of R_4+5_ bare; distal angle (bend of vein M_1_) obtusely curved (in *M. lactineala* Pape, 1997, vein M_1_ fading within the membrane); hind tibia with equally strong dorsal, anterodorsal and posterodorsal preapical setae.

##### Distribution.

Afrotropical, Oriental and Palaearctic regions.

### Key to Chinese species of genus *Morinia* Robineau-Desvoidy, 1830 (males)

Note: except *M. crassitarsis* Villeneuve, 1936.

**Table:** 

1	Eyes bare	**2**
–	Eyes with sparse hairs	**3**
2	Parafacial with 1–2 rows of hairs; hind tibia with 1 av s, 2 pd s; no notch between lateral lobes of ST5; surstyli two-segmented	***M. proceripenisa* Feng, 2004**
–	Parafacial with 3–5 rows of long setae; hind tibia without av s, with 4–5 pd s; ST5 normally shaped; surstyli undivided	***M. setifrons* Xue & Du, 2022**
3	Fore tibia without av s; ST5 with a membranous, semicircular median protuberance between lateral lobes	***M. zhenhang* Fu, Xue & Cui, sp. nov**.
–	Fore tibia with 2 av s; ST5 normally shaped	***M. piliparafacia* Fan, 1997**

#### Morinia zhenhang

Taxon classificationAnimaliaDipteraPolleniidae

Fu, Xue & Cui sp. nov.

2F8F4C6C-70C2-521C-BC6B-2C91A50CF6E9

https://zoobank.org/9AB3FAE3-C452-4B8E-A95D-305BEAF3CA28

Figs 1, 2, 3, 4, 5, 6, 7, 8, 9, 25, 26

##### Vernacular name.

(振航墨粉蝇)

##### Type material.

***Holotype*** • male (HIST), Beihang University campus, Beijing, China, 40°9'4"N, 116°15'55"E, 4. VI. 2025, 20 m, leg. Zhenshuang Huang. ***Paratypes*** • 2 male (HIST), same data as holotype; 1 male (SEMCAS), Huanglong, Shaanxi Province, China, 10. VIII. 1983, leg. data absent.

##### Diagnosis.

Eyes with short, sparse microsetae; parafacial bare; ac s 1 + 1, ia s 0 + 2, pra s 1, sa s1, kept s 1:1, prepst s 1, and prepm s 1; basicosta black; ventral hairs of costa not reaching subcostal break; ST5 with a membranous, semicircular median protuberance between lateral lobes; anterior processes of the basal part of paraphallus present.

##### Description.

**Figure 1.**
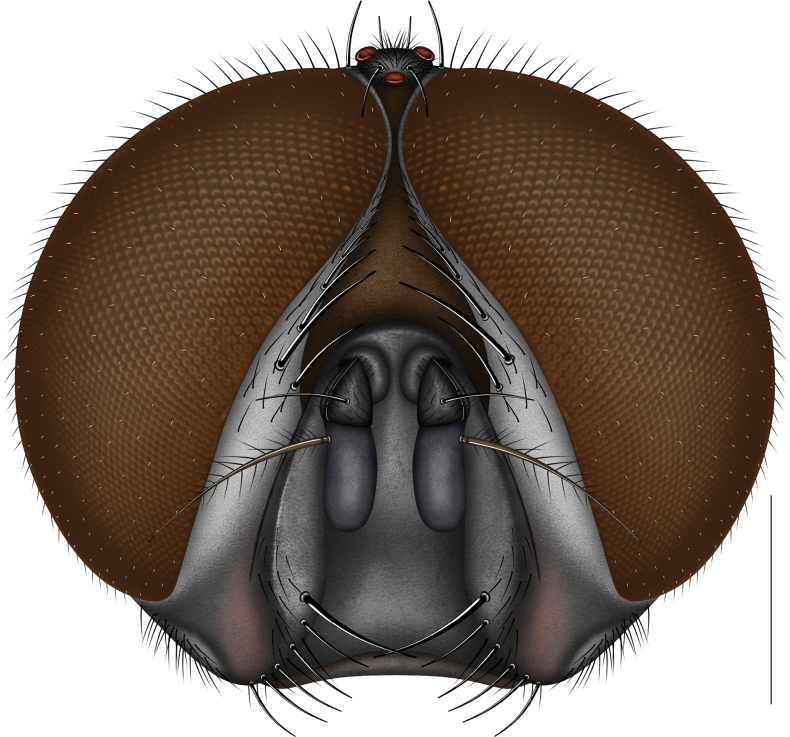
Head of *Morinia zhenhang* sp. nov., male (holotype), frontal view. Scale bars: 1.0 mm.

**Figure 2.**
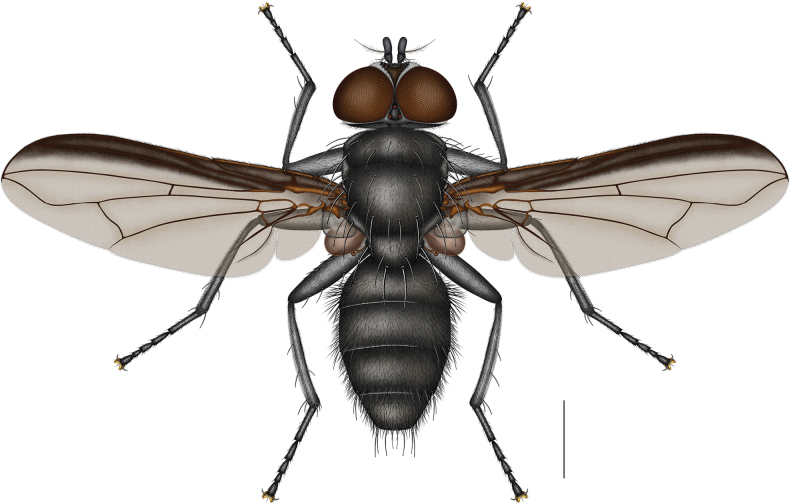
*Morinia zhenhang* sp. nov., male (holotype), habitus. Scale bars: 1.0 mm. Chaetotaxy of all left femora and tibiae in anterior view, and of the right in posterior view.

**Male. *Head*** (Figs 1, 2). Holoptic, eyes with short, sparse microsetae, upper ommatidia relatively enlarged; frontal vitta dark brown, narrowed medially to a thin line; frons index about 0.02; iv s 1 (weak), povt s 1, pavt s 1; fronto-orbital plate and parafacial black, with silvery-white pruinosity, former with 1–2 rows of black hairs, latter bare; fr s 6, on lower about 3/5 of frons; genal groove dark, slightly reddish brown at junction of ptilinal fissure and facial ridge; facial ridge black, with hairs on lower 1/3; lunule, face, and facial carina black; facial carina weak; gena anterior part with silvery-white pruinosity, hairs black; gena height about 0.16 × eye height; beard black; antenna black, postpedicel about 2.0 × length of pedicel; arista dark brown, plumose, basal 1/4 thickened, dorsal branches more numerous than ventral branches, apical 1/3 bare; palpus black.

***Thorax*** (Figs 2, 25, 26). Black, with black hairs; anterior and posterior thoracic spiracles dark; prosternum, anatergite, katatergite, metapleuron, and metasternum bare, remaining pleura with black hairs; suprasquamal ridge bare, anterior, posterior, and tympanic tufts absent.

***Chaetotaxy*** (Figs 2, 25, 26). Ac s 1 + 1, dc s 2 + 3, ia s 0 + 2, pprn s 2, ph s 1:0, pr s 1, pra s 1, sa s 2, pal s 2, sc s 1:2; npl s 2; kept s 1:1, prepst s 1, prepm s 1.

***Wings*** (Fig. 2). Grayish-brown, transparent; costal margin distinctly dark infuscated, A_1_ vein slightly infuscated, other veins without distinct infuscation; tegula, and basicosta black; subcostal sclerite brown, with black microsetae; ventral hairs of costa extending beyond the humeral break but not reaching the subcostal break, covering about 1/4 of the distance between them; radial node bare dorsally and ventrally; cell r_4+5_ open, opening narrow, about 0.3 × length of r–m; distal angle (bend of vein M_1_) obtusely curved, posterior portion slightly curved toward cell r_4+5_; dm–m slightly S-shaped; upper calypter dirty white, with dark margin; lower calypter narrow, brownish; halter brownish.

***Legs*** (Fig. 2). Black; fore femur with complete posterodorsal and posteroventral rows of setae, fore tibia with 1 ad s; mid femur with posteroventral row of setae on basal half only, mid tibia with 1 ad s, 1 pd s, 2 p s, and 1 v s; hind femur with anterodorsal setae from base to apex, anteroventral setae from near base to near apex, and posteroventral setae on the basal part, hind tibia with 2 ad s, 1 av s, and 2 pd s.

***Abdomen*** (Figs 2, 8, 25, 26). Ovoid, ground colour black; T3 without median marginal setae; T4 with marginal setae; ST1–5 with black hairs; ST5 with a membranous, semicircular median protuberance between lateral lobes (Fig. 8).

**Figures 3–9.**
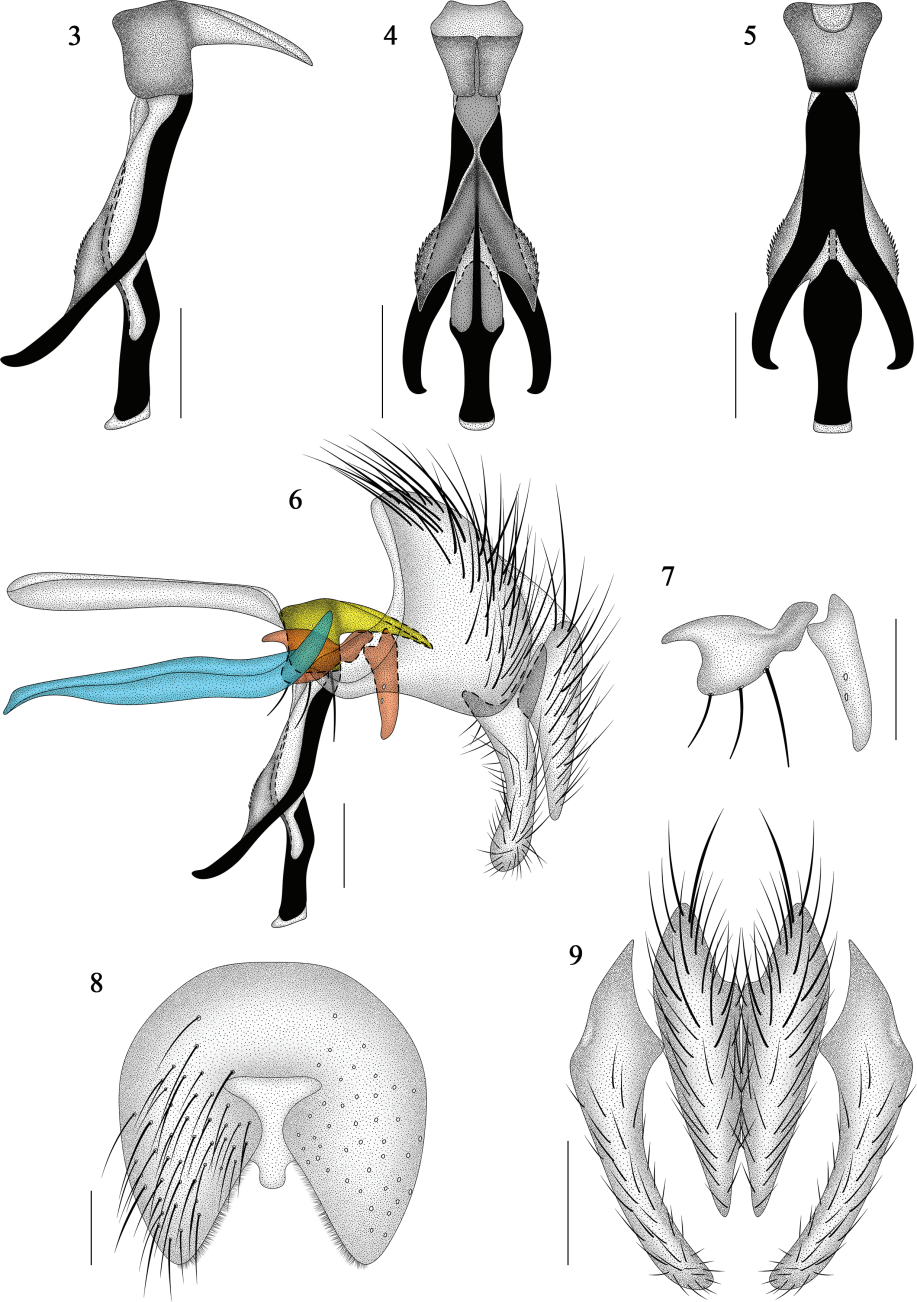
*Morinia zhenhang* sp. nov., male (holotype). **3**. Phallus, lateral view; **4**. Phallus, dorsal view; **5**. Phallus, ventral view; **6**. Terminalia, lateral view; **7**. Pregonite (left) and postgonite (right); **8**. ST5, ventral view; **9**. Cerci and surstyli, ventral view. Scale bars: 0.2 mm. In 8, the bluish, reddish, and yellowish colours represent the hypandrium; pregonite and postgonite; and basiphallus and epiphallus, respectively, from outside to inside.

***Terminalia*** (Figs 3–7, 9). In ventral view, cerci gradually taper from base to apex, with apices not extending beyond those of the surstyli (Fig. 9); surstyli with base slightly wider than apex (Fig. 6); hypandrium nearly equal in length to phallapodeme; pregonite bearing 3 setae (Fig. 8); postgonite with 2 setae (absent in type materials); distal process of paraphallus slightly incurved, its length extending far beyond the lateral hypophallic lobe (Figs 3–6); hypophallic middle piece fused at the junction of the hypophallic lobes; apex of hypophallic middle piece heavily sclerotized and expanding laterally and fused ventrally; acrophallus membrous and thin (Figs 3–6); lateral hypophallic lobes distinctly sclerotized, with many small spines.

**Female**. Unknown.

##### Measurements.

Male. Body length 4.6–4.8 mm.

##### Etymology.

Named for the fly collector Zhen-Shuang Huang (“Zhen”, meaning “inspiring”) and the location of type specimens, Bei-Hang University (“Hang”, meaning “flying in sky”).

##### Distribution.

China (Beijing, Shaanxi).

#### Genus Pollenia

Taxon classificationAnimaliaDipteraPolleniidae

Robineau-Desvoidy, 1830

EE92A772-6D57-56CF-9FFC-6CB779CD9EE7

Pollenia Robineau-Desvoidy, 1830: 412. Type species: Musca rudis Fabricius, 1794, by original designation.

##### Diagnosis.

Adults of this genus can be recognized by the combination of the following characters: ia s 0–1 + 2; hind tibia with the posterodorsal preapical setae indistinct, or with the anterodorsal, dorsal, and posterodorsal preapical setae subequal in length; parafacial setulose; body metallic or non-metallic in coloration, abdomen rarely yellowish. Lower calypter usually broad; subcostal sclerite usually bearing a bundle of long black or yellowish setae among the micropubescence.

##### Distribution.

Afrotropical, Australasian, Nearctic, Oriental and Palaearctic regions.

### Key to Chinese species of genus *Pollenia* Robineau-Desvoidy, 1830

Modified from Fan (1997).

**Table:** 

1	Basicosta dark	**3**
–	Basicosta yellowish or yellowish-brown	**6**
2	Abdomen shiny black, without pruinosity	***P. alajensis* Rohdendorf, 1926**
–	Abdomen black in ground colour, with pruinosity	**4**
3	Costal margin with dark infuscation	**5**
–	Costal margin without dark infuscation	***P. ernangshanna* Feng, 2004**
4	Mid tibiae with 1 pd s and 2 p s; gena height about 0.48 × eye height; cercal prongs diverging; ♀ frontal vitta width about 2.5 × fronto-orbital plate width	***P. shaanxiensis* Fan & Wu, 1997**
–	Mid tibiae pd s absent, with 3 p s; gena height about 0.41 × eye height; cercal prongs not diverging; ♀ frontal vitta width about 3.0 × fronto-orbital plate width	***P. yuensis* Fu, Xue & Cui, sp. nov**.
5	Bases of all femora with yellowish hairs	***P. huangshanensis* Fan & Chen, 1997**
–	Bases of all femora without yellowish hairs	**7**
6	♂ hind tarsi anteroventrally with a row of developed pecten; ♀ hind tarsi anteroventrally with a row of slightly developed pecten	***P. pectinata* Grunin, 1966**
–	Hind tarsi anteroventrally without developed pecten	**7**
7	Gena height approximately 0.33 × of eye height; palpus brownish-yellow	***P. sichuanensis* Feng, 2004 (**♀ **unknown)**
–	Gena height approximately equal to eye height; palpus brown to black	**8**
8	Underside of wing with tuft of pale setae at intersection of subcosta and humeral crossvein	***P. pediculata* Macquart, 1834**
–	Underside of wing without tuft of pale setae at intersection of subcosta and humeral crossvein	***P. rudis* (Fabricius, 1794)**

#### Pollenia yuensis

Taxon classificationAnimaliaDipteraPolleniidae

Fu, Xue & Cui sp. nov.

534AF240-1ECA-5E3C-88C1-AD2B6EF7F9B4

https://zoobank.org/9CDBC1D9-3E7D-4BD3-8E7C-02A049E28323

Figs 10, 11, 12, 13, 14, 15, 16, 17, 18, 19, 20, 21, 22, 23, 24, 27, 28

##### Vernacular name.

(豫粉蝇)

##### Type material.

***Holotype*** • male (HIST), Huanglianshu, Jiyuan, Henan Province, China, 35°15'03"N, 112°04'15"E, VII. 2023, 1430 m, leg. Tong Fu. ***Paratypes*** • 2 male (HIST), Beihang University campus, Beijing, China, 40°9'16"N, 116°15'44"E, 28. VI. 2025, 40 m, leg. Zhenshuang Huang; • 10 male (HIST), same data as holotype; 8 female (HIST), same data as holotype; • 1 male (SEMCAS), same data as holotype; • 1 female (SEMCAS), same data as holotype.

##### Diagnosis.

Dark infuscation along the costal margin and wing veins; outer ph s absent; costa hairy below only to junction with sc vein; basicosta dark; mid tibiae pd s absent, with 3 p s; phallus with a median midventral hypophallic lobe.

##### Description.

**Figures 10, 11.**
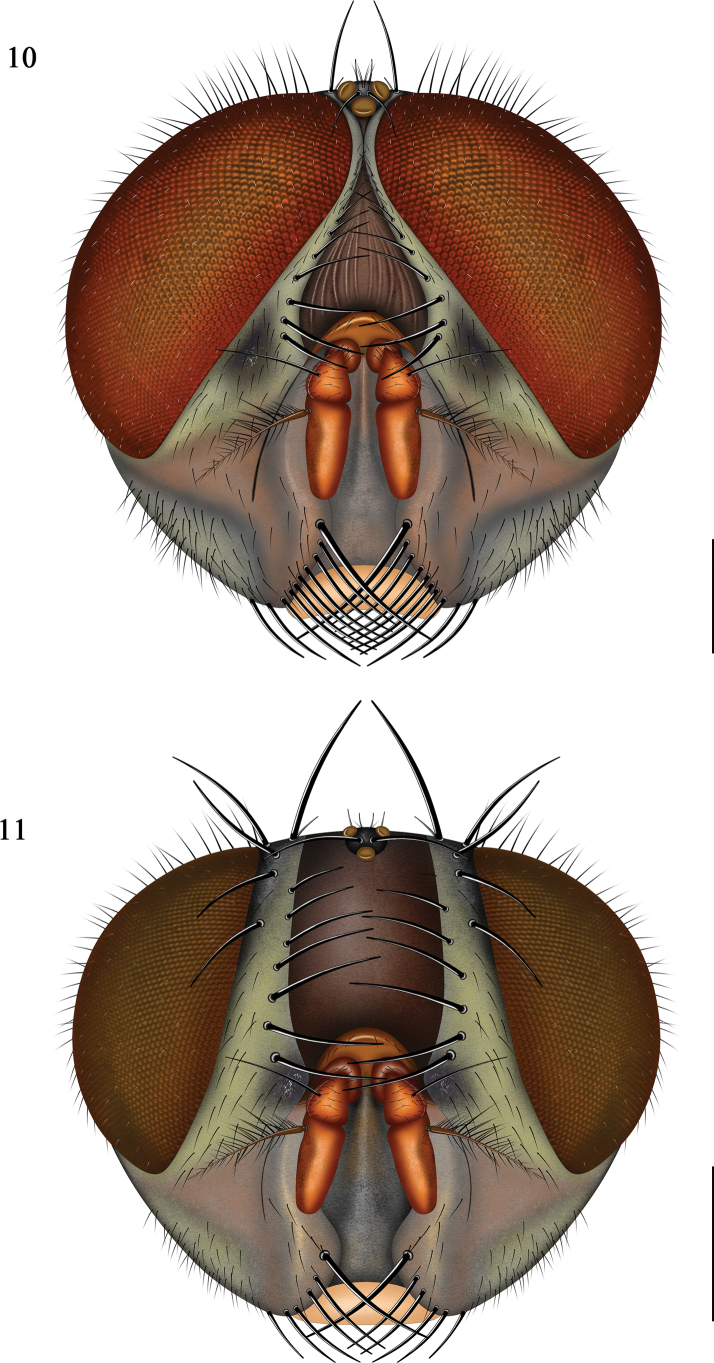
Head of *Pollenia yuensis* sp. nov. **10**. Male (holotype), frontal view; **11**. Female (paratype), frontal view. Scale bars: 1.0 mm.

**Figures 12–13.**
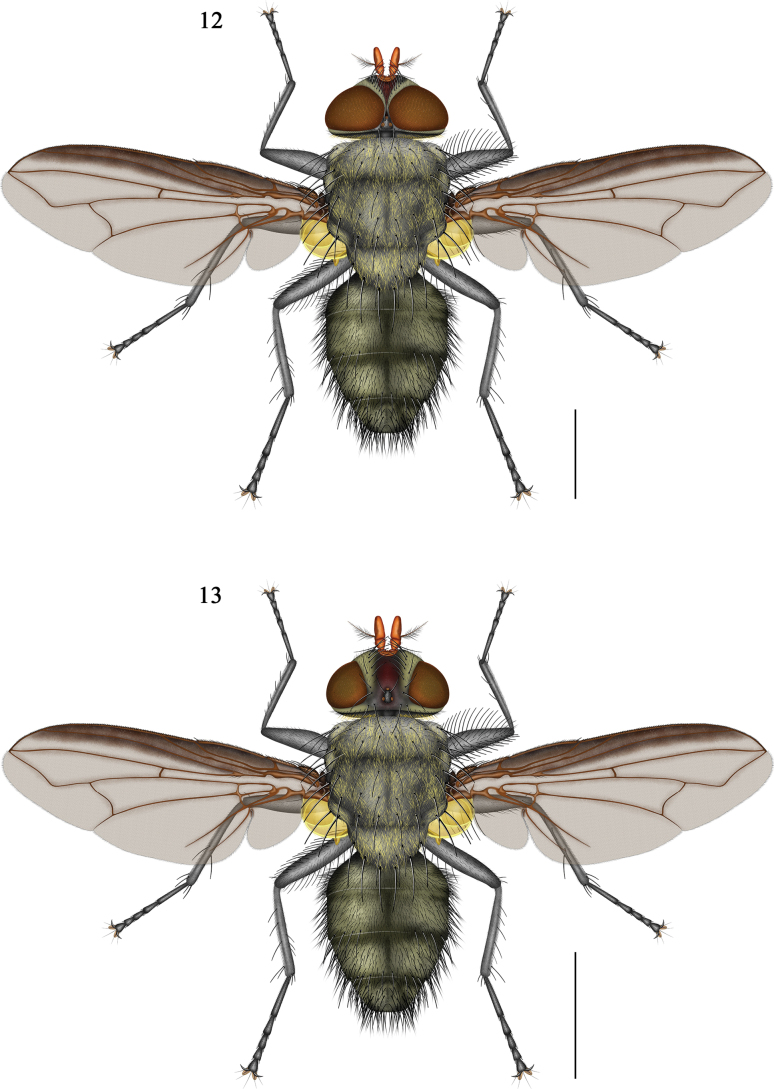
*Pollenia yuensis* sp. nov. **11** Male (holotype), habitus; **12**. Female (holotype), habitus. Scale bars: 2.0 mm. Chaetotaxy of all left femora and tibiae in anterior view, and of the right in posterior view.

**Male. *Head*** (Figs 10, 12). Holoptic, eyes with short, sparse microsetae, upper ommatidia relatively enlarged; frons slightly narrower than anterior ocellus, frontal vitta brownish with longitudinal wrinkles, narrowed to a line or absent at the narrowest point; iv s 1, povt s 1, pavt s 1; fronto-orbital plate, parafacial and gena black with grayish-yellow pruinosity, all setae black, fr s 10 (upper 3 pairs hair-like); parafacial with a variable dark patch, about 1.8 × width of postpedicel; genal groove reddish brown; gena height about 0.41 × eye height; lunule reddish, bearing a few hairs; facial ridge reddish brown with hairs on lower 1/3; facial carina sharp and narrow, reddish apically, slightly widened on lower 1/3; lower margin of face slightly reddish; antenna reddish-orange, postpedicel darker anteriorly, about 2.1 × as long as wide, and 1.8 × the length of pedicel; arista reddish brown, plumose, thickened at basal 1/4, longest branches slightly longer than postpedicel width, dorsal branches denser than ventral; postgena with black hairs near mouthparts, yellowish hairs elsewhere; occiput with mostly yellowish hairs, except marginal rows of black hairs; palpus yellowish-brown.

***Thorax*** (Figs 12, 27, 28). Black, postpronotal lobe and anterior margin of prescutum covered with gray pruinosity; prescutum with 1 pair of fine black submedian stripes; scutum with 1 median black stripe that disappears in posterior view, where 1 submedian black stripe appears on each side, both thicker than those on the prescutum; thorax with sparse golden crinkly hair-like setae, scutellum with slightly denser setae; both anterior and posterior thoracic spiracles yellowish; prosternum bare; anepisternum and katepisternum bear golden crinkly hair-like setae and black hairs; postalar wall, anepimeron, and meron near posterior thoracic spiracle with only golden crinkly hair-like setae; katatergite bare; anatergite with black hairs only near suprasquamal ridge; suprasquamal ridge bare, anterior, posterior, and tympanic tufts absent.

***Chaetotaxy*** (Figs 12, 27, 28). Ac s 2 + 3, dc s 2 + 3, ia s 1 + 2 (1^st^ postsutural ia s sometimes absent), pprn s 3, ph s 1:0 (rarely 1:1), pr s 1, pra s 1, sa s 2, pal s 2, sc s 1:3 (rarely 2:3); npl s 2; kept s 1:1, prepst s 1, prepm s 1.

***Wings*** (Fig. 12). Grayish-brown, transparent; costal margin with dark infuscation, but infuscation along wing veins faint; tegula black; basicosta dark brown; subcostal sclerite yellowish, bearing yellowish setae and occasionally a few black setae; costa vein hairy ventrally only from base to junction with sc vein; radial node with about 3 setae dorsally and ventrally; cell r_4+5_ open, narrow, about 1/3–1/4 length of r-m vein; distal angle (bend of vein M_1_) obtusely curved, posterior portion slightly bent toward r_4+5_ cell; dm-m vein slightly S-shaped; upper calypter yellowish, lower calypter slightly brownish; halter yellowish.

***Legs*** (Fig. 12). Black; fore femur with complete rows of setae on posterodorsal and posteroventral surfaces, fore tibia with 4–5 ad s and 2 pv s; mid femur with a row of setae only on the basal half of the posteroventral surface, mid tibia with 1 ad s, 3 p s, and 1 v s; hind femur with complete rows on anterodorsal and anteroventral surfaces, and a row on basal half of posteroventral surface, hind tibia with 3–4 ad s, 2–3 av s, and 3–4 pd s; pulvilli with dark brownish sclerotized claw marks centrally.

***Abdomen*** (Figs 12, 19, 27, 28). Ovoid, ground colour black, with indistinct greyish-yellow tessellate pruinosity on tergites; T3–T5 with indistinct median black stripe, more distinct in posterior view; T3 without median marginal setae; T4 with marginal setae; ST1–5 with black hairs, ST1 additionally with yellowish hairs; ST5, see Fig. 19.

**Figures 14–20.**
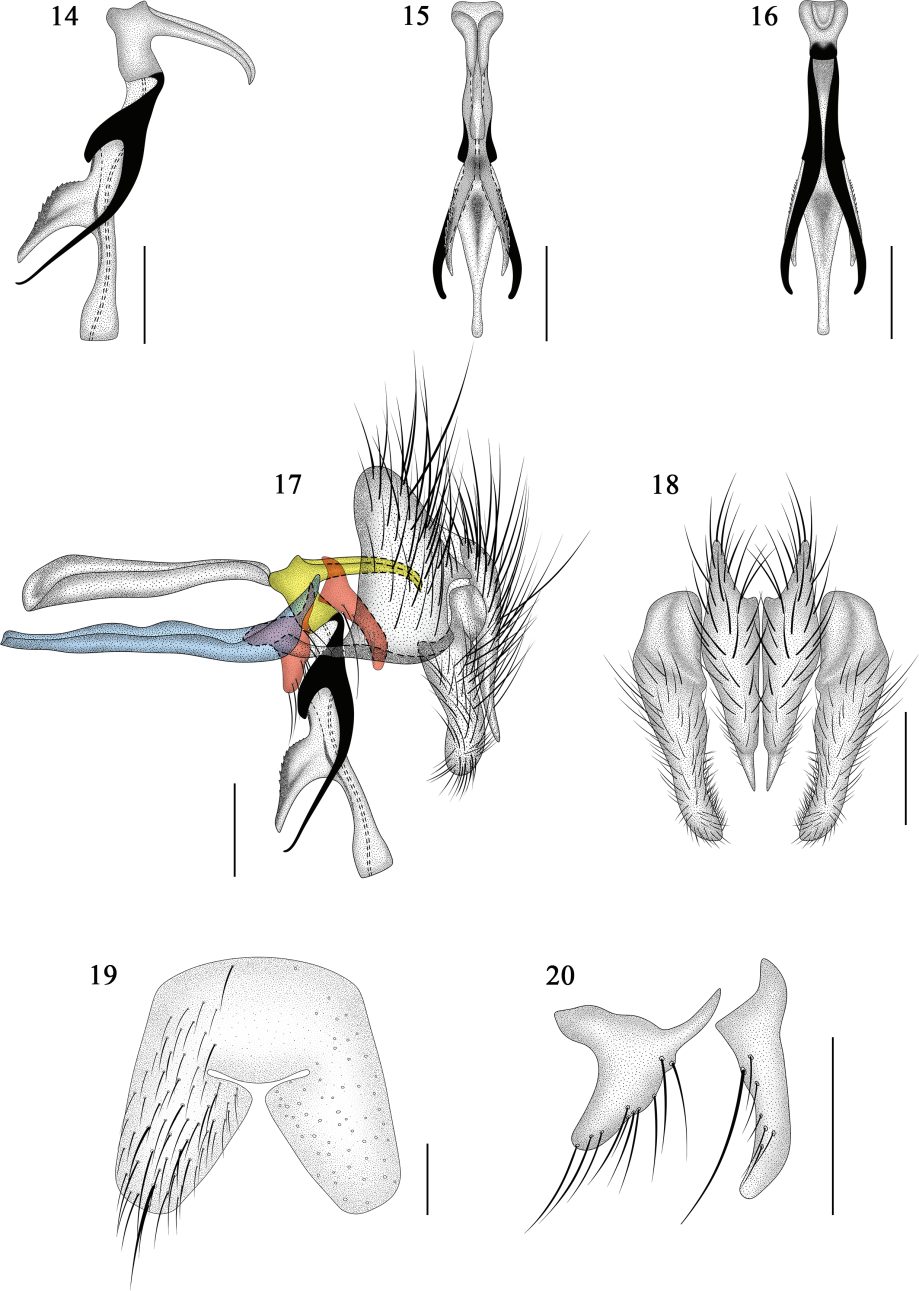
*Pollenia yuensis* sp. nov. male (holotype). **14**. Phallus, lateral view; **15**. Phallus, dorsal view; **16**. Phallus, ventral view; **17**. Terminalia, lateral view; **18**. Cerci and surstyli, ventral view; **19**. ST5, ventral view; **20**. Pregonite (left) and postgonite (right). Scale bars: 0.2 mm. In 17, the bluish, reddish, and yellowish colours represent the hypandrium; pregonite and postgonite; and basiphallus and epiphallus, respectively, from outside to inside.

***Terminalia*** (Figs 14–18, 20). In ventral view, cercal prongs diverging at base, while apical portions are converging or parallel, but never diverging, and never extending beyond the tip of surstyli (Fig. 18); in lateral view, the cercal prongs are everted (Fig. 17); basal half of surstyli are slightly widened, with tip are rounded; hypandrium is slightly longer than phallapodeme; pregonite with several setae (Fig. 20); postgonite with 1 long seta and a few short setae; hypophallic middle piece indistinct at anterior processes of base of paraphallus (Figs 14, 17); lateral hypophallic lobes with small spines along the dorsal margin and with a central sclerotization (Figs 14–17); distal processes of paraphallus are rounded at the tip, slightly curved inward, and slightly extend beyond the hypophallic ventral process.

**Female** (Figs 11, 13). Frons index approximately 0.43; width of frontal vitta about 3.0 × the width of parafacial, without longitudinal wrinkle; parafacial covered with grayish-yellow pruinosity; poc s indistinct, pc orb s 2, fr s 6–8. Other characters same as male.

**Figures 21–24.**
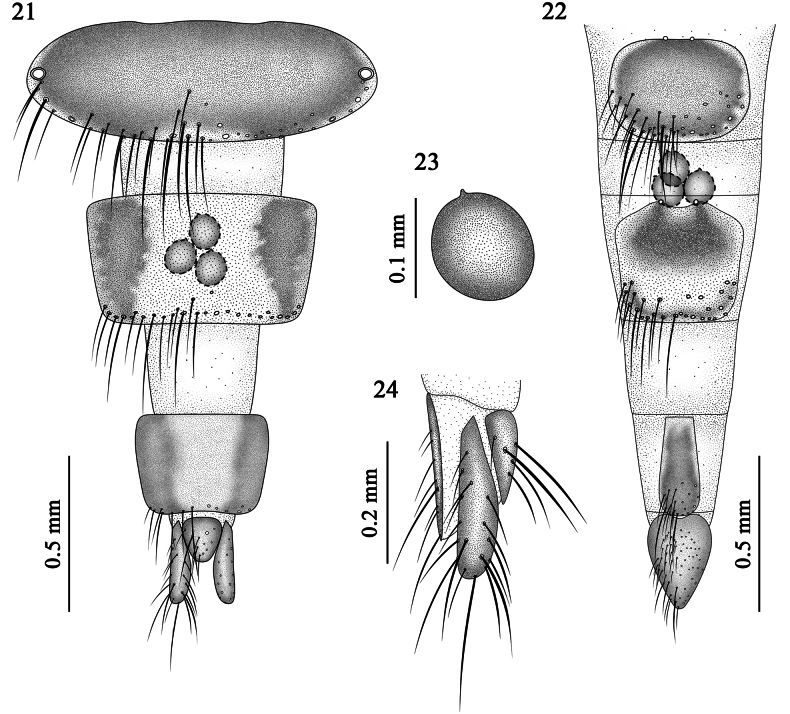
*Pollenia yuensis* sp. nov. female (paratype). **21**. Terminalia, dorsal view; **22**. Terminalia, ventral view; **23**. Spermatheca; **24**. Proctiger and cercus, lateral view.

***Terminalia*** (Figs 21–24). T6 elongate-oval, with weak sclerotization along lateral and ventral margins (Fig. 21); ST6 rounded-oval, with weak marginal sclerotization (Fig. 22); T7 inverted trapezoid, slightly more sclerotized near lateral base; central and lateral areas weakly sclerotized; ST7 dorsal margin convex dorsally, slightly concave medially, basal half and area near ventral margin slightly more sclerotized, central and marginal areas weakly sclerotized; T8 inverted trapezoid, strongly sclerotized laterally, weakly sclerotized medially; ST8 elongate-strip-like, ventral margin rounded, with weak marginal sclerotization; epiproct and hypoproct subtriangular; cerci rod-shaped (Figs 21, 24); spermatheca pyriform (Figs 21–23).

**Figures 25–28.**
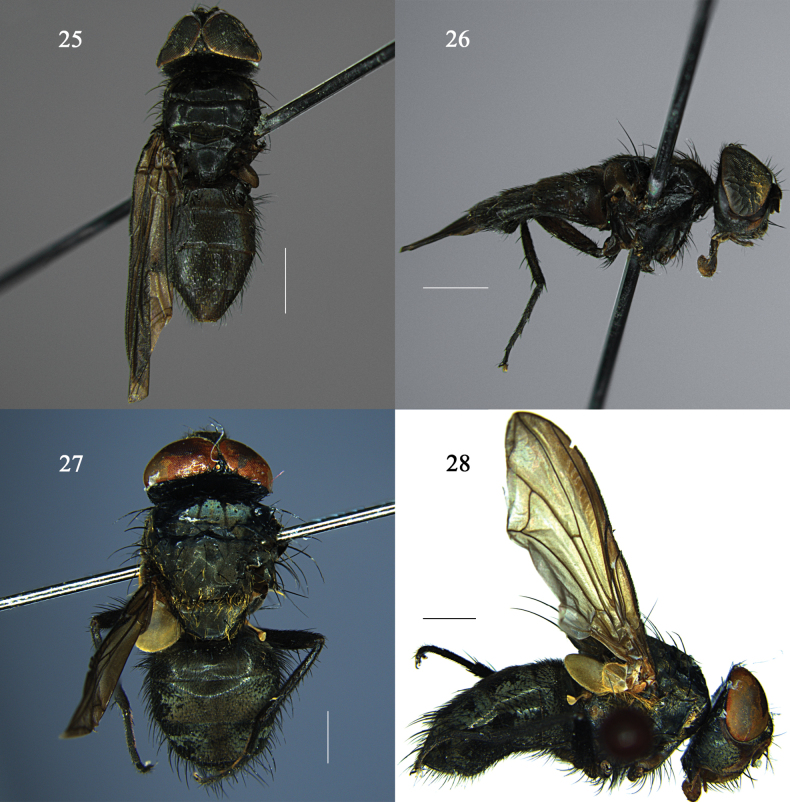
*Morinia zhenhang* sp. nov. male (holotype), and *Pollenia yuensis* sp. nov. (holotype). **25**. *M. zhenhang*, habitus; **26**. *M. zhenhang*, lateral view; **27**. *P. yuensis*, habitus; **28**. *P. yuensis*, lateral view. Scale bars: 1.0 mm.

##### Measurements.

Male. Body length 6.7–8.4 mm. Female. Body length 4.5–9.0 mm.

##### Etymology.

The species epithet *yuensis* refers to “Yu”, the Chinese abbreviation for Henan Province, the type locality of this species.

##### Distribution.

China (Henan, Beijing).

## Discussion

Male specimens of *Morinia* from the Afrotropical Region exhibit marked differences compared to those from the Oriental and Palaearctic regions: Afrotropical males are dichoptic, whereas males from the Oriental and Palaearctic regions are holoptic. Furthermore, the three species of *Morinia* previously recorded from China (*M. piliparafacia*, 2500 m; *M. proceripenisa*, 2670 m; *M. setifrons*, 2140 m) are all distributed at high-altitude areas, while *M. zhenhang* sp. nov. represents the first record of *Morinia* from a low-altitude (20 m) area in China (Fan 1997; Feng 2004; Xue and Du 2022). Clearly, this genus occurs at both low- and high-altitude in China.

*Morinia crassitarsis* was not included in this study. *Morinia crassitarsis* is known from Sichuan, China (Gisondi et al. 2020). In Villeneuve’s (1936) original description, the information was quite brief, but he mentioned that the first tarsomere of the fore leg is wider in the female than that of the male, which may be a useful diagnostic character. However, the females of the other four Chinese *Morinia* species are still unknown.

## Supplementary Material

XML Treatment for Morinia

XML Treatment for Morinia zhenhang

XML Treatment for Pollenia

XML Treatment for Pollenia yuensis

## Additional information

### Use of AI

No use of AI was reported.

### Author ORCIDs

Tong Fu https://orcid.org/0009-0006-0531-6874

Yawen Xue https://orcid.org/0009-0009-2687-9125

Jianxin Cui https://orcid.org/0000-0002-0963-1496

### Data availability

All of the data that support the findings of this study are available in the main text.
